# Modeling Preeclampsia In Vitro: Polymorphic Variants of STOX1-A/B Genes Can Downregulate CD24 in Trophoblast Cell Lines

**DOI:** 10.3390/ijms232415927

**Published:** 2022-12-14

**Authors:** Marei Sammar, Clara Apicella, Peter Altevogt, Hamutal Meiri, Daniel Vaiman

**Affiliations:** 1 Prof. Ephraim Katzir’s Department of Biotechnology Engineering, Braude College of Engineering, 51 Snunit St., Karmiel 2161002, Israel; 2 Institute Cochin, U1016, INSERM, UMR 8504 CNRS, Paris-Descartes Université, 75014 Paris, France; 3 Skin Cancer Unit, DKFZ and Department of Dermatology, Venereology and Allergology, University Medical Center Mannheim, Ruprecht-Karl University of Heidelberg, Theodor-Kutzer-Ufer 1–3, 68167 Mannheim, Germany; 4 Hylabs, Rehovot and TeleMarpe, 21 Beit El St., Tel Aviv 6908742, Israel

**Keywords:** CD24, preeclampsia, placental-derived immortalized cells, JEG-3 cells, BeWo cells, STOX1-A/B, in vitro preeclampsia models, immune tolerance

## Abstract

CD24 is a mucin-like immunosuppressing glycoprotein whose levels increase during pregnancy and decrease in the syncytio- and cytotrophoblasts in early and preterm preeclampsia. We used two modified cell lines that mimic in vitro features of preeclampsia to identify if this phenomenon could be reproduced. Our model was the immortalized placental-derived BeWo and JEG-3 cell lines that overexpress the STOX1 A/B transcription factor gene that was discovered in familial forms of preeclampsia. BeWo and JEG-3 cells stably transduced with the two major isoforms of STOX1-A/B or by an empty vector (control), were propagated, harvested, and analyzed. CD24 mRNA expression was determined by quantitative real-time polymerase nuclear chain reaction (qRT-PCR). CD24 protein levels were determined by Western blots. In STOX1-A/B overexpressing in BeWo cells, CD24 mRNA was downregulated by 91 and 85%, respectively, compared to the control, and by 30% and 74%, respectively in JEG-3 cells. A 67% and 82% decrease in CD24 protein level was determined by immunoblot in BeWo overexpressing STOX1-A/B, respectively, while the reduction in JEG-3 cells was between 47 and 62%. The immortalized BeWo and JEG-3 cell lines overexpressing STOX1-A/B had reduced CD24. Although both cell lines were affected, BeWo appears to be more susceptible to downregulation by STOX-1 than JEG-3, potentially because of their different cell origin and properties. These results strengthen the in vivo results of reduced CD24 levels found in early and preterm preeclampsia. Accordingly, it implies the importance of the reduced immune tolerance in preeclampsia, which was already demonstrated in vivo in the STOX1-A/B model of preeclampsia, and is now implied in the in vitro STOX-1 model, a subject that warrants further investigations.

## 1. Introduction

The pathogenesis of preeclampsia and other hypertensive pregnancy disorders are poorly understood, despite a substantial worldwide research effort and the high burden of maternal and neonatal morbidity associated with this condition [1,2,3]. In particular, the role of genetic variants as determinants of disease susceptibility has not been discovered [4], although family history [5] and ethnic origin are major prior risk factors for the syndrome [6].

In 2005, van Dijk et al. [7] showed that storkhead box 1 (STOX1), a transcription factor belonging to the enlarged FOX family, has a strong association with genetic polymorphisms located inside the open reading frame of this gene in patients with familial forms of preeclampsia. Clinical studies have shown that in subsets of patients with the Y153H variant of STOX1 there was systemic endothelial dysfunction, hypertension, and proteinuria. Despite some later studies that raised questions on the composition of the originally used cohort, subsequent studies by Tyberghein et al. [8] and van Dijk et al. [9] showed the STOX1 impact on placental cell migration/invasion mechanisms. Doridot et al. [10] have demonstrated the role of STOX1 in balancing oxidative/nitrosative stress.

Studies have shown that STOX1 has two major isoforms: STOX1-A (the most complete, encompassing, in particular, a DNA-binding domain and a transactivator domain, 989 amino acids), and STOX1-B, coding for a 227 amino acid polypeptide, which does not encompass the transactivator domain [7,11]. Vaiman and Miralles [12] hypothesized that the two isoforms could compete for the same DNA binding site(s) thus triggering different responses and that their imbalance could result in placental damage and hypertensive pregnancy disorders. Recently, it was revealed by exome sequencing that there are rare variants of STOX1 associated with HELLP syndrome, a preeclampsia subtype with serious hepatic complications [13].

In 2013, Doridot et al. [14] developed a transgenic mouse model expressing STOX1 at the fetoplacental level. During pregnancy, these mice developed hypertension and proteinuria, often combined with fetal growth restriction, which is reversed by aspirin and also by alpha-1 microglobulin [14,15]. These preeclampsia features found in the STOX1 mouse model were also accompanied by changes in the placental vascular and extracellular matrix, and are linked to impaired electron transfer in the mitochondria of placental cells [10,14,16]. In some cases, STOX1 expression, particularly in extravillous trophoblasts, was linked to the activation of the uteroplacental renin–angiotensin system [17,18]. Using in vitro and ex vivo approaches, van Dijk et al. [7,9] showed that the risk allele (Y153H) of the preeclampsia susceptibility gene STOX1 negatively regulates trophoblast invasion by upregulating the cell–cell adhesion protein alpha-T-catenin (CTNNA3) [19].

In vitro models were also developed in immortalized placental cell lines transfected with STOX1-A and STOX1-B [20] using BeWo and JEG-3 cell lines; in these models, there is a >20–30-fold overexpression of STOX1-A and a >6–10-fold overexpression for STOX1B. Transcriptome analysis showed that in these cells, STOX1 elicits the down or upregulation of 12.5% of the genes [11,20], mimicking the modification of gene expression observed in the preeclamptic placenta including the genes for Endoglin, Syncytins-1 and 2, human chorionic gonadotrophin (hCG), Progesterone, LGALS13 (PP13), and LGALS14 (PP14), among others. This gene list includes preeclampsia’s main risk prediction markers or modulators [5,16,20,21,22].

The potential impact of STOX1 on immune suppression (pivotal for immunotolerance during pregnancy) has not yet been specifically explored. Among the suppressor proteins involved in these pathways is CD24 which has been shown to be an important factor in the immune suppression of these pathways. CD24 is a marker of regulatory B cells [23] that are involved in increasing immunotolerance, especially in the context of grafts [24].

CD24 is a small (27 amino acids) protein attached to the membrane via a glycosylphosphatidylinositol (GP-I) anchor [25,26]. It has many potential glycosylation sites for N- and O-linked carbohydrate binding, rendering the molecule structurally similar to mucins [27]. CD24 binds to Siglec-10, and together they form a strong immunosuppressive axis [28]. The binding of the Siglec10–CD24 axis was demonstrated to be an important immune checkpoint for immune tolerance in mouse autoimmune models [29,30]. Recently, CD24 was identified as an immune modulator in cancer cells inhibiting the phagocytic potential of macrophages, as was shown by Barkal et al. [31].

Recombinant CD24, in the form of CD24 fusion protein bound to the fragment crystalized (Fc) part of immunoglobulins of the antibodies (CD24-Fc), was recently demonstrated to be a promising drug for blocking over-shooting immune reactions (“the cytokine storm”) in SARS-CoV-2 infections [32]. CD24-containing exosomes were also found in a clinical trial to reduce the symptoms and the severity of COVID-19 [33], indicating a pharmacological link between immune suppression and CD24 in different diseases.

In previous publications, our group has studied placental CD24 and found that its first-trimester expression is linked to the glandular epithelial cells of the uterine glands and other decidual cells [34]. As was previously reported [27,28], it was also found that the protein is co-expressed with Siglec-10, mainly in the close vicinity of the invasive extravillous trophoblasts [34]. In another study, we used qRT-PCR analysis to show a significant increase in CD24 expression from the first and early second trimester to the third trimester and term delivery [35]. In contrast to the normal course of pregnancy, in cases of early (before 34 weeks gestation), and preterm (before 37 weeks gestation) preeclampsia, the level of CD24 mRNA is reduced [35] compared to cases of term delivery and preterm delivery (before 37 weeks gestation). Such a reduction in CD24 mRNA was also found before [36]. Moreover, in addition to mRNA reduction, it was found that in cases of early and preterm preeclampsia, immunohistochemistry labeling by CD24 was reduced in the syncytio-and-cytotrophoblasts, compared to preterm and term controls [35]. In contrast, a higher expression of CD24 protein was found in term preeclampsia cases compared to term and preterm controls [35].

In the present study, we evaluated the impact of STOX1A/B overexpression in BeWo and JEG-3 cells on CD24 expression. We found a reduced expression level of CD24 mRNA and protein in BeWo cells, and to a lower extent in JEG-3 cells.

## 2. Results

### 2.1. CD24 Expression in Cell Line Transfected by the Polymorphic Variants of STOX1-A and B

**Figure 1:**
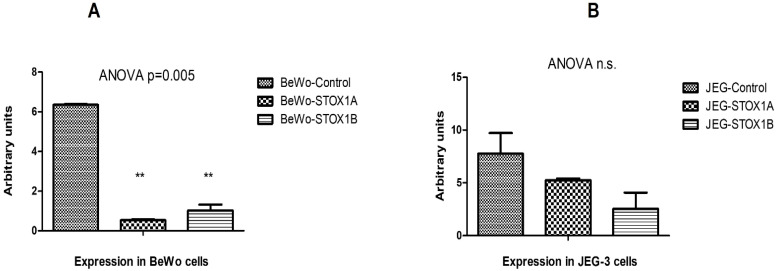
Downregulation of CD24 expression in (**A**) BeWo and (**B**) JEG-3 cells overexpressing STOX1-A and STOX1-B variants. The qPCR experiments were conducted in duplicates using, as a normalizer, the geometric average of the ΔCT of the two reference genes (HPRT and YWHAZ). The error bars are SEM. The two asterisks in A represent the individual one-way ANOVA significant level of each STOX1 variant compared to the control (** *p* < 0.01). The lower degree of downregulation of CD24 in the JEG-3 vs. BeWo cells could be due to the different origins and features of these two placental-derived cell lines.

We applied qRT-PCR analyses to study the CD24 expression in BeWo and JEG-3 cells overexpressing STOX1-A or STOX1-B compared to those encompassing the empty vector (control). Measured against two reference genes (HPRT and WYHAZ), a reduced CD24 expression was found for both STOX1 isoforms in BeWo and JEG-3 (Figure 1A,B). The expression of CD24 (2^−ΔΔCT^) was massively and significantly reduced in the BeWo cell line for STOX1-B [85%] and STOX1-A [91%] (Figure 1A) compared to the control (** *p* < 0.01). The downregulation of CD24 in the JEG-3 cell line was more moderate [74%] in STOX1-B and [30%] in STOX1-A-overexpressing cells (Figure 1B).

The 2^−ΔΔCT^ of CD24 expression is presented for the cell line overexpressing STOX1-A/B relative to the mock empty control vector (100%), all standardized against the housekeeping genes HPRT and YWHAZ; A—BeWo cells, B—JEG-3 cells. The analysis used one-way ANOVA, followed by a post-hoc test: Student–Neumann–Keuls. The stars correspond to these post hoc tests, i.e., comparing controls vs. STOX1-A and vs. STOX1-B, separately.

### 2.2. Bioinformatic Analysis of the CD24 Promoter

A FIMO analysis [36] was carried out on the position −2000 to +500 of the CD24 promoter obtained from the EPD database [37] for STRE1 (CATTTCACGG) and STRE2 (GGTGYGGAMA), as identified in Ducat et al. (2020) [11]. A unique hit was found, but with an FDR (q-value) of 0.204 (Table 1).

### 2.3. Western Blot Analysis

Western Blot analysis of CD24 showed that the protein level was decreased in both cell lines overexpressing STOX1-A and B.

**Figure 2:**
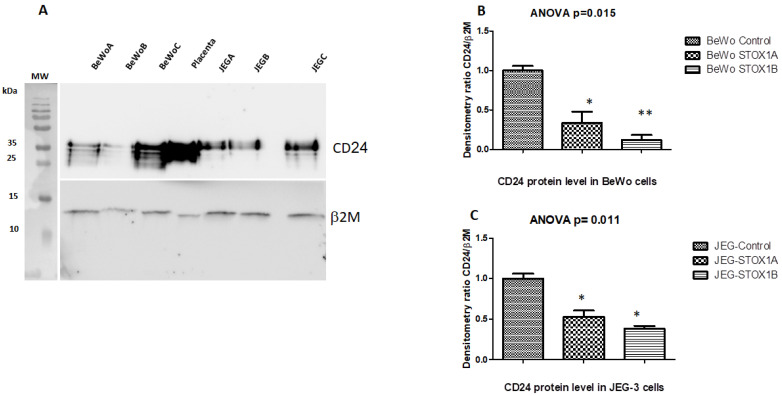
Western blot analyses of the CD24 expression in STOX1-A/B-stably transfected BeWo and JEG-3 cells. (**A**) Western blot analysis of the CD24 protein expression in trophoblast cell transfectants by STOX1-A/B and mock control of BeWo cells and of JEG-3 cells. CD24 was analyzed by Western blotting with a specific anti-CD24 monoclonal antibody (upper panel). Βeta2-microglobulin was used as a control for loading equal amounts of proteins on the gel (lower panel). (**B**,**C**) Semiquantitative densitometric assessment of CD24 and Β2-microglobulin bands was performed using ImageJ software. The data represents density at two exposure time points. The intensity of the CD24 bands was normalized to β2-microglobulin as a control for the equal loading of proteins. The results have a similar pattern in three different repeats. The statistic is based on one-way ANOVA followed by a post hoc test: Student–Neumann–Keuls. The stars were obtained by comparing controls vs. STOX1-A and STOX1-B, separately (* *p* < 0.05 and ** *p* < 0.01). The figure is representative of the Western blot experiments that were conducted three times and showed a similar pattern of CD24 reduction. The histogram error bars are SEM.

The total amount of CD24 in BeWo was significantly decreased by 67% (*p* = 0.015) when they overexpressed STOX1 A, and by 82% (*p* = 0.008) when they overexpressed STOX1-B compared to the control BeWo-C cells (Figure 2B).

In the JEG-3 cells, the reduced CD24 level was more moderate when compared to the reduction in the mRNA levels. In JEG-3 cells, CD24 was significantly reduced by 47–62% (*p* = 0.011) in both STOX1-A and B compared to the control (Figure 2C).

The lower degree of the downregulation of CD24 in JEG-3 vs. BeWo could be due to the capability of BeWo cells to go to the end of their differentiation program, since they can fuse, while JEG-3 may be blocked in the status of nondifferentiated trophoblasts, and are possibly less sensitive to fusion signaling modulation.

The downregulation of CD24 was found in early and preterm preeclampsia [35]; the higher impact of STOX1-B over STOX1-A may be related to their differential features as transcription factors in gene expression, as it was demonstrated earlier for other proteins [11].

## 3. Discussion

CD24 appears to be one of the major actors in immune tolerance. It is a cell-surface protein marking B-reg cells, a subset of B cells that suppress immune reactions [38], as was already found in organ transplantation [38,39]. Since gestation can be defined as a semi-allograft, it would be desirable to identify whether B-reg-cell-mediated immune modulation is involved in a successful pregnancy.

The importance of STOX1 variants in preeclampsia was initially shown among Dutch familial cases [7,9], and also recently in the Turkish population [40]. On the contrary, no clear link to preeclampsia was found among Korean women [41] or in a number of European ancestries. Familial aspects of the disease are debatable [42], although a family history of preeclampsia is a worldwide known prior risk factor to develop preeclampsia [6].

Here, we attempt to make a connection between the overexpression of STOX1 and its link to reduced immune tolerance in pregnancy. For this purpose, we used JEG-3 and BeWo cells that overexpressed STOX1. BeWo cells have similarities with the villous trophoblasts (they can be induced to generate syncytia, but do not express HLA-G) [21,24]. JEG-3 cells are more similar to extravillous trophoblasts since they are highly proliferative but do not present syncytial fusion [21,24,43,44,45]. Therefore, these two cell types could serve as models, albeit imperfect ones, for two major types of placental cells. In vivo, the overexpression of STOX1 reproduced a few preeclamptic features [10,11], such as oxidative/nitrosative stress, and in previous in vitro studies, members of our team have shown that it is associated with membrane damage and impaired syncytialization.

Our main findings in this study are (1) reduced CD24 mRNA expression in BeWo and JEG-3 cells overexpressing STOX1-A/B, and (2) decreased level of CD24 protein in BeWo and to a lower extent in JEG-3 cells overexpressing STOX1-A/B. Altogether, our data demonstrate that there is a downregulation of CD24 in the overexpression of STOX1-A/B in trophoblast cell lines and that this effect is stronger in BeWo than in JEG-3 cells. Regarding the mechanism of deregulation, we attempted to identify STOX1 binding sites in the CD24 promoter. A putative STRE2 is found, however, STRE2 is not bound directly by STOX1 [11], but probably by a partner, since in most cases where a direct regulation occurs, both STRE1 and STRE2 are present together and STOX1 binds exclusively to STRE1 [11]. In summary, we cannot exclude an indirect interaction of STOX1 on the CD24 promoter that could explain the deregulation.

In early pregnancy, villous trophoblasts come into close contact with various types of maternal blood vessels, and with immune cells from the decidua, they are exposed to different oxygen concentrations throughout pregnancy (hypoxia, then normoxia) [21,43]. In this study, we found that the overexpression of STOX1 leads to the reduced expression of CD24, which is anticipated to negate immune tolerance. This suggests a novel link between the STOX1 gene in pregnancy and CD24 in a direction that may suppress the immune tolerance offered by CD24 (i.e., the link between overexpression of the STOX1 transcription factor could reduce the expression of CD24 and impair the CD24-Siglice10 axis which may impair immune tolerance the in the trophoblast cells). The physiological importance of CD24 expression by nonimmunological cells has not been clarified. However, we noticed that the expression level of CD24 in JEG-3 cells is similar to that which has already been found for the most expressed genes in this model. It implies that there is physiological importance of CD24 in trophoblasts, as was already shown for B cells. The verification of the importance of CD24 in JEG-3 cells, however, warrants further investigation.

The extravillous trophoblasts (modelized here by JEG-3) are formed early in pregnancy. They come into contact with a number of cells from the uterine wall, including decidual stromal cells and some immune cells, e.g., uNK cells. Since HLA-G is expressed in those cells, their need for control by the STOX1–CD24 axis is less essential than for villous trophoblasts [21,24,43,44,45]. In addition, during pregnancy, the extravillous trophoblasts do not encounter varying oxygen concentrations. When the extravillous trophoblasts invade the uterine wall, it occurs under standard oxygen concentrations. Thus, the electron transfer, which involves STOX1, may not be essential to the trophoblasts’ function in comparison to the function of villous trophoblasts. If so, the overexpression of STOX1-A/B may not be so damaging to the JEG-3 cell line that resembles the extravillous trophoblast layer. This is consistent with the differential efficacy of STOX1 overexpression in decreasing CD24 protein levels, as we have found for JEG-3 in this study. Interestingly, another protein related to immune tolerance is PP13 or LGALS13. This protein is also expressed in the syncytiotrophoblasts and its level was increased in BeWo cells stimulated to syncytialization [5]. The expression of PP13 was not found in extravillous trophoblasts [45], and consistently, its level in JEG-3 cells is also very low [46].

We overexpressed the STOX1 A/B transcription factor gene that was discovered in familial forms of preeclampsia in immortalized placental cell lines. The results presented here are solid, despite being limited in scope, and may drive more extensive research by us and by other groups.

In accordance with our results, we propose an additional aspect to the role of STOX1 in increasing the risk of preeclampsia through the regulation of immune suppression. Since STOX1 suppresses CD24 which confers immune tolerance, this study indicates that there is an additional pathway for STOX1 involvement in the development of preeclampsia. Accordingly, the following process is delineated: In normal pregnancy, the blastocyst is implanted into the uterine wall and the invading trophoblasts enter the decidua. These cells express cell-surface proteins of a paternal origin; hence, a normal pregnancy is dependent on immune tolerance to enable the maintenance of the gestation. The development of immune tolerance involves certain sets of proteins including HLA-G and others; we propose that CD24 is one of them. Although our culture model of overexpression of STOX1 in BeWo and JEG-3 cells does not mimic major aspects of preeclampsia such as hypertension and proteinuria, it could still serve in the study of certain other molecular aspects of the disorder such as the reduction in the expression and in the level of proteins that are involved in immune tolerance. Since, in the past, we showed that in preeclampsia cases there is a reduction in the expression of CD24, our results in this in-vitro model enable us to consider the reduced level of CD24 as an additional aspect of reduced immune tolerance in preeclampsia. This direction may lead to exploring the supplement of CD24 as a therapeutic agent to fight preeclampsia.

**Figure 3:**
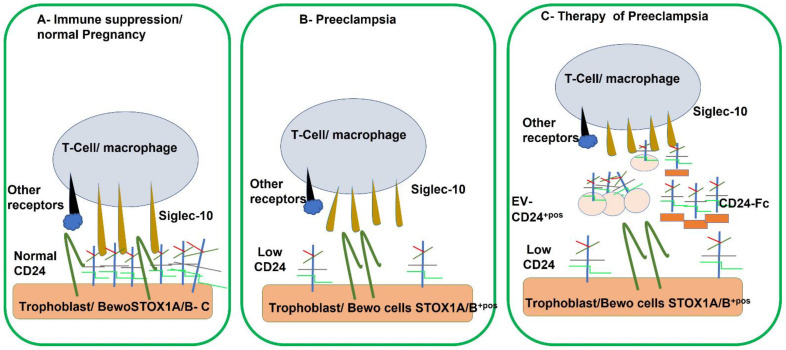
A schematic model of the potential role of CD24 in a normal and in preeclampsia is depicted to indicate how CD24 replenishment could help fighting preeclampsia. (**A**) In normal pregnancy, the immunosuppressive process involves the formation of a complex made of CD24, Siglec-10 and the control STOX1 A/B (STOX1-A/B—C) on the surface of BeWo/trophoblast cells. The complex triggers the inhibitory signals and promotes immunosuppression. (**B**) in preeclampsia there is a reduced amount of CD24-Siglec 10 due to the over expression of the mutated variants of sTOX1 A/B on the surface of the BeWo cells, leading to the loss of immune suppression. (**C**) When replenishment the cultured cells with extracellular vesicles (EV) that have a rich expression of CD24 on their surface, (EV-CD24^+pos^) or with CD24-Fc soluble molecule, the BeWo/trophoblast regain immune tolerance and recover from preeclampsia.

A model of CD24 action in normal and preeclamptic pregnancy is proposed in Figure 3. Part A shows the interaction of Siglec-10 on immune cells with the expression of CD24 on the placental trophoblasts that were invading the decidua (or in our model, with BeWo cells). Such activity will result in an active immunosuppressive process during normal pregnancy (Figure 3A). When CD24 expression is limited such as in preeclampsia or BeWo trophoblasts transfected with STOX1A/B, the immunosuppression and immunotolerance fail to protect the pregnancy (Figure 3B).

Therefore, in this hypothetic model (Figure 3C), the replenishment of CD24 deems necessary for maintaining immune suppression during pregnancy. Therefore, a putative treatment with either CD24-Fc molecule or extracellular vesicles enriched with CD24 [32,33] could serve as a potential therapy to cure preeclampsia.

## 4. Materials and Methods

### 4.1. Antibodies

The monoclonal antibody (mAb) clone SWA11 was used for the detection of CD24 [47,48]. This mAb is specific for CD24 and reacts with the leucine–alanine–proline (LAP) motif in the protein core, as shown by peptide inhibition studies [48]. In addition, SWA11 exhibits specific binding to CD24-transfected cells but not to a vector control [47].

### 4.2. Plasmid Preparation

The STOX1-A and STOX1-B constructs were a generous gift of Dr. C.B. Oudejans (VU University Medical Center, De Boelelaan 1117, 1081 HV, Amsterdam, The Netherlands). The ORF was isolated and subcloned in the pCMX expression vector and re-sequenced. Three mutations were found (ARC in 1658 (GAA = GluRGCA = Ala), a deleted T in 2948 (which creates an early stop codon), and the initiation codon (ACG (THR) instead of ATG (MET)) and corrected by site-directed mutagenesis. Hence, we obtained a pCMX vector containing the coding region of STOX1-A. STOX1-B was also re-sequenced from the relevant plasmid.

### 4.3. Transfection

Cell culture and stable transfection JEG-3 choriocarcinoma cells were seeded in DMEM medium (Gibco) supplemented with 10% FBS and 1% penicillin/streptomycin at a concentration of 106 cells per T25 culture flask. Passages of the cells were made between 5 to 10 min before processing.

At the time of transfection, the cells were at 60% confluence. The cells were transfected with the PGK-neo expression vector [25] using Lipofectamine 2000 Reagent (Invitrogen, Waltham, MA, USA), according to the manufacturer’s recommendations. Four (4) μg of pCMX-STOX1-A or 4 μg empty pCMX together with 0.4 µg of PGK-neo per T 25 cm^2^ culture dish with Opti-MEMH I Reduced Serum Medium. This plasmid ratio is known to ensure a co-transfection by both plasmids when the cells become resistant. The cells were passaged at 1:10 dilution into a selective medium 72 h post-transfection. The selection was continuously applied using Geneticin (G-418) (Invitrogen) at 500 mg/mL concentration for approximately 3 weeks. Resistant clones (nine cell lines transfected with pCMX-STOX1-A and three cell lines transfected with empty pCMX) were grown individually in the continual selection and used for further analysis or frozen in DMSO. Similarly, BeWo cells were stably transfected except that the cells were cultivated in an F12 medium (Life Technologies, Carlsbad, CA, USA) supplemented with FBS and antibiotics. JEG-3 cells were supplemented with 5-to-15 passages before processing.

Samples of mRNA were prepared in triplicates from two independent cell culture experiments and RT-PCR was performed using the MMLV kit from Invitrogen. The expression of STOX1-A or B was then assessed by qRT-PCR. The cells and their detailed preparation and selection were published previously [11,19]. They were all cultivated in an adequate medium complemented with Geneticin G-418 at 500 µg/mL [11]. BeWo-A and BeWo-B overexpressed STOX1-A and STOX1-B (20 and 6-fold, respectively), and BeWo-C was their related control (with the empty G-418 resistance plasmid). For JEG-3 cells, AA6 (JEG-3A) overexpressed STOX1-A (~30 fold), B10 (JEG-3B) overexpressed STOX1-B (~4–6 fold), and BD3 (JEG-3C) was the related control cell line.

### 4.4. RT-PCR Amplification of CD24 mRNA

RNA preparation was carried out in cells by direct lysis using the Trizol–Chloroform method, following the manufacturer’s protocol.

Reverse transcription was carried out using the MMLTV reverse transcriptase kit from Invitrogen (Thermo-Fisher, Bd Sébastien Brant, Parc d’Innovation, France).

The expression of the CD24 gene was quantified by TaqMan RT-PCR utilizing the Applied Biosystem StepOne Plus cycler (Applied Biosystems, Austin, TX, USA) and TaqMan Gene Expression Assay with primer and probe sets (Applied Biosystems) for CD24 (Hs02379687_s1).

HPRT and WYHAZ (ABI, Branchburg, NJ, USA) were used to standardize the expression level. The relative amount of CD24 was calculated by employing the comparative CT method (2^−ΔΔCT^) [49]. Amplification was performed for the JEG-3 and BeWo cells transfected by STOX1-A or STOX1-B polymorphic variants compared with the empty expression vector used as a mock control.

### 4.5. Western Blot

BeWo and JEG-3 stable transfectant cells were trypsinized, pelleted, washed twice in PBS, and resuspended for 1 h at 4 °C in RIPA (5 mM Tris HCl pH 7.6, 150 mM NaCl, 1% NP-40, 1% sodium deoxycholate, 0.1% SDS) containing a cocktail of protease inhibitors (100× Thermo-Fisher, DTT 50 mM, and PMSF 50 mM). Insoluble material was removed by centrifugation (20,000× *g*, 4 °C), and the protein concentration of the soluble fraction was determined by a BCA reagent (Pierce, Rockford, IL, USA). Placental lysate samples were aliquoted and stored at −70 °C until use.

For Western blot analysis, 50 µg of total protein lysates were separated on 12.5% SDS-PAGE at 130 volts for 2 h. in Tris-Glycine, 2% SDS buffer, at room temperature; proteins were then electro-transferred to nitrocellulose membranes at 70 volts in Tris-Glycine buffer at 4 °C for 2 h. The transfer was evaluated by Ponceau red staining. Membranes were blocked in PBS with 0.1% Tween 20 and 5% defatted milk (Regilait) for 1 h, then rinsed thrice in PBS-Tween 0.1%. After blocking free binding sites, membranes were probed with the anti-CD24 mAb SWA11 and anti-β-globin antibodies (0.1 µg/mL) overnight at 4 °C. Bound immune complexes were detected by horseradish peroxidase-conjugated rabbit anti-mouse IgG and developed using an ECL detection kit (Biological Industries, Beit Haemek, Israel). The β2M was used as an internal control for the equal loading of proteins. Signals were developed using chemiluminescence and were captured using an imager (Bio-Rad, Hercules, CA, USA).

### 4.6. Statistics

In the different experiments, statistics were based on one-way ANOVA, followed by a post hoc test: the Student–Neumann–Keuls test [50].

## 5. Conclusions

The downregulation of CD24 mRNA expression and its total protein levels were determined in placental-derived immortalized cell lines BeWo and JEG-3 cells that were overexpressing STOX1 A/B. STOX1-A appears more potent than STOX1-B in suppressing CD24 expression, and although both BeWo and JEG-3 cells were affected, BeWo appears more susceptible to downregulation by STOX-1 than JEG-3, potentially due to the differential cell origin. CD24 has already been found to convey immune suppression in cancer. Here, its reduced expression and protein levels were determined in the in vitro model of preeclampsia which is similar to the reduced CD24 found in early and preterm preeclampsia [35]. This may be another indication of the link between preeclampsia, a major pregnancy complication, and immune rejection, which has yet to be further explored.

## Figures and Tables

**Table 1:** A FIMO analysis on the position of CD24 promotor on the position −200 to +500 was extracted from the EPD database for the STRE1 sequence (CATTTCACGG) and STRE2 sequence (GGTGYGGAMA).

Motif ID	Alt ID	Sequence Name	Strand	Start	End	*p*-Value	q-Value	Matched Sequence
1	GGTGYGGAMA	CD24Promoter	-	2466	2475	4.35 × 10^−5^	0.204	GGTGTGGAAT

## Data Availability

Not applicable.
